# Bacteriomic Profiling of Branchial Lesions Induced by *Neoparamoeba perurans* Challenge Reveals Commensal Dysbiosis and an Association with *Tenacibaculum dicentrarchi* in AGD-Affected Atlantic Salmon (*Salmo salar* L.)

**DOI:** 10.3390/microorganisms8081189

**Published:** 2020-08-05

**Authors:** Joel Slinger, Mark B. Adams, James W. Wynne

**Affiliations:** 1 CSIRO Agriculture and Food, Aquaculture Program, Bribie Island, QLD 4507, Australia; 2 Institute of Marine and Antarctic Studies, University of Tasmania, Launceston, TAS 7250, Australia; Mark.Adams@utas.edu.au; 3 CSIRO Agriculture and Food, Aquaculture Program, Hobart, TAS 7000, Australia; James.Wynne@csiro.au

**Keywords:** Neoparamoeba, AGD, Bacteria, Atlantic salmon, bacteriome, gill microbiota, *Tenacibaculum*, Tenacibaculosis

## Abstract

Amoebic gill disease is a parasitic condition that commonly affects marine farmed Atlantic salmon. The causative agent, *Neoparamoeba perurans*, induces a marked proliferation of the gill mucosa and focal superficial necrosis upon branchial lesions. The effect that amoebic branchialitis has upon gill associated commensal bacteria is unknown. A 16S rRNA sequencing approach was employed to profile changes in bacterial community composition, within amoebic gill disease (AGD)-affected and non-affected gill tissue. The bacterial diversity of biopsies with and without diseased tissue was significantly lower in the AGD-affected fish compared to uninfected fish. Furthermore, within the AGD-affected tissue, lesions appeared to contain a significantly higher abundance of the *Flavobacterium*, *Tenacibaculum dicentrarchi* compared to adjunct unaffected tissues. Quantitative PCR specific to both *N. perurans* and *T. dicentrarchi* was used to further examine the co-abundance of these known fish pathogens. A moderate positive correlation between these pathogens was observed. Taken together, the present study sheds new light on the complex interaction between the host, parasite and bacterial communities during AGD progression. The role that *T. dicentrarchi* may play in this complex relationship requires further investigation.

## 1. Introduction

The outer gill surface of teleost fish represents a unique and dynamic landscape where microbial antigens within the external milieu attempt to invade the mucosal interface, whilst the host immune system attempts to overcome these continuous insults [1,2]. Furthermore, collateral damage during this conflict can lead to profound changes in the commensal microbial community, which may ultimately contribute to complex disease pathologies. Previous research has shown that the seawater environment contains up to 10^7^ organisms per milliliter [3], and therefore, represents a rich source of microbes that, under certain conditions, can have negative effects on the host. A delicate balance exists between commensal and opportunistic pathogens, however under certain conditions such as disease or poor environmental conditions this microbial balance can be lost, leading to a dysbiosis where opportunistic species dominate [4].

Several examples exist where the commensal host-associated microbiota is significantly impacted as a result of opportunistic pathogen infection. The commensal microbiomes of Asian seabass (*lates calcarifer*) and largemouth bronze gudgeon (*Coreius guichenoti*) demonstrated an appreciable loss of richness and diversity when affected by tenacibaculosis and furunculosis respectively [5,6]. Similarly, the microbiome of salmon skin was observed to be in a state of imbalance at the interface of the pathogen and host during infection with ectoparasitic sea lice (*Lepeophtheirus salmonis*) [7]. Regular aquaculture operations can also influence bacterial communities, with soy-based dietary additives [8] and husbandry practices such as seawater transfer and handling [9,10] having significant impacts on the internal and external microbiota in respective in vivo studies with Atlantic salmon. Commensal probiotic treatments can provide some microbial resilience to finfish, as observed in both black molly (*Poecilia sphenops*) and Arctic char (*Salvelinus alpinus*), when a probiotic additive was seen to lessen the detrimental impact of two significant pathogenic bacterial species, *Vibrio anguillarum* and *Flavobacterium psychrophilum* respectively [11,12].

The fish gill is a structurally and functionally complex organ that responds rapidly to adverse circumstances. Amoebic gill disease (AGD) is a parasitic gill condition affecting Atlantic salmon (Salmo salar) aquaculture globally. The aetiological agent of the condition is Neoparamoeba perurans [13], a marine free-living amphizoic amoeba, which upon attachment to the gill surface elicits clinical responses including grossly visible white mucoid patches upon the gills, respiratory distress, hypernatremia, inappetence and mortality if untreated [14,15]. Multifocal gill lesions are dominated by hyperplasia of the respiratory mucosa with variable inflammatory infiltration [16,17,18,19,20,21,22]. While the host response to AGD is well characterized, the impact (and interactions) that AGD may have with the commensal bacterial community is largely unknown to date. Previous studies have investigated the relationship between specific candidate bacterium and AGD development. A culture independent assessment of AGD affected farm salmon demonstrated a possible link between amoebic infection and *Psychroserpens* taxa [23]. Similarly, experimentally induced AGD lesion incidence was exacerbated in the presence of *Winogradskyella* sp. in Atlantic salmon [24].

It may be possible that *N. perurans* directly interacts with the microbial population on the gill, either by eliciting localized agents responsible for lesion formation, or by fulfilling a role as a potential vector to harbor and transport bacterial taxa to the gill surface. Indeed, complex and often symbiotic relationships exist between amoeba and bacteria, that may ultimately effect pathogeneses in the host. In vitro studies have shown that *N. perurans* produces cytolytic extracellular products (ECPs) [25,26] which are likely to underly necrotic fenestrations observed at amoebae attachment sites upon gill lesions of AGD affected fish [27,28,29]. Pathogenic amoebae such as *Entamoeba histolytica* also utilize the production of proteases to enhance host cellular degradation once attached to the tissue surface [30]. Further to this, it has been shown that these processes have enhanced disease severity when *Entamoebae* trophozoites utilize known pathogenic bacteria as a feed source [31] that augment cytolytic effects. The attachment mechanism of *N. perurans* to gill surfaces may also be bacteria-assisted, as with other pathogenic amoeba species such as *Acanthamoeba* (causative agent of eye keratitis) favoring attachment sites in the presence of key bacteria which are known to produce specific cytopathic proteases [32].

Techniques in culture independent bacterial profiling now exist to give greater clarity and understanding of how commensal host microbiota on the gill surface interface responds to amoebic (*N. perurans*) insult. Here, we hypothesize that changes in branchial surface morphology and cellularity at lesion sites associated with infection by *N. perurans* may alter the bacteriomic profile of infected gills in Atlantic salmon. The aims of this study were therefore to investigate whether the diversity and richness of branchial microbial communities could be altered by experimentally induced AGD, investigate whether these indices vary discretely between diseased and non-diseased gill tissues and ascertain whether particular bacterial taxa are prominent in AGD-affected fish in vivo.

## 2. Materials and Methods

All animal procedures were approved under application (#2018-09) by the Queensland CSIRO Animal Ethics Committee under the guidelines of the Australian Code of Practice. Fish used for this work were humanely killed (immersion bath of 100 ppm AQUI-S anaesthetic) prior to sampling. Seawater for this experiment was sourced from a plant delivering a constant supply of filtered (~40 µm), ozonated (100 gO_3_·h^−1^), UV treated (80 mJ·cm^2^) and chilled (~15 °C) seawater to the laboratory via offshore spear pumps.

### 2.1. Amoebic Challenge and 16S rRNA Bacterial Community Analysis

#### 2.1.1. Experimental Challenge with *Neoparamoeba Perurans*

Following habituation (7 d) in a flow-through seawater tank (5000 L), 50 Atlantic salmon smolt (naïve to AGD) were transferred to a 1000 L seawater (35 ppt) flow-through tank with temperature maintained at 15 ± 0.5 °C, pH at 7.8 ± 0.1 and dissolved oxygen maintained between 90–100% saturation. Fish were fed daily to satiation using a commercial 3 mm pellet (Nutra, Skretting P/L, Cambridge, Australia). The remaining 500 fish were purposed for use in an AGD challenge trial [33] and were exposed to wild-type gill associated trophozoites of *N. perurans* as follows. A sufficient volume of water (taken from a recirculating system containing AGD affected Atlantic salmon) was introduced to the holding system in sufficient volume to achieve a final concentration of 100 *N. perurans*.L^−1^ (1 h static exposure). The concentration of infective amoebae was determined from 1 L samples of system water centrifuged at 4000 *g* to concentrate cells into a final volume of 10 mL. Wild-type *N. perurans* stock concentration was then enumerated by averaging repeated trophozoite counts (*n* = 10) on a haemocytometer. A subset of 33 Atlantic salmon were taken from the exposure tank and stocked into a single 500 L flow-through seawater tank. Fish were subsequently maintained at 15 ± 0.5 °C, pH 7.8 ± 0.1, 35 ppt and dissolved oxygen saturation between 90–100%. The infected fish were fed daily to satiation with a commercial diet (Nutra 3 mm, Skretting P/L, Australia) until sampling at 21 days post-infection (dpi).

#### 2.1.2. Tissue Biopsy Sampling

**Figure 1:**
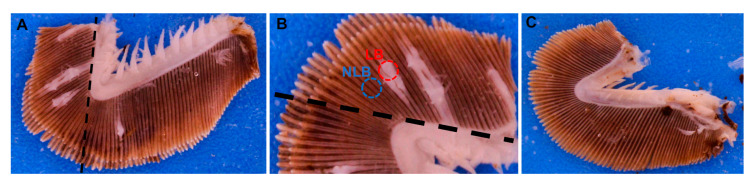
(**A**)—Example of AGD affected RH3 hemibranch, and (**B**)—subset of (**A**), showing biopsy punch samples obtained from AGD originating gross gill lesions (red) and adjacent unaffected tissue (blue) located in the dorsal region (above dotted black line). (**C**)—depicts hemibranch from AGD naïve smolt, with no visible gross pathology.

For AGD affected fish (*n* = 10), the dorsal region of the third holobranch on the right-hand side of the gill basket (RH3) was examined for viable AGD lesions (Figure 1). A gill filament biopsy of up to 3 hyperplastic AGD lesions was excised a using sterile one-use biopsy punch (2 mm diameter) methodology adapted from prescribed methods in [16,34], and placed into a 1.5 mL tube containing 1 mL RNAlater solution. An area adjacent to each lesion of unaffected (normal) gill filaments were then excised using a new punch and placed into a separate 1.5 mL tube containing 1 mL RNAlater solution. RNALater preserved samples were stored in the freezer at −20 °C until DNA extraction.

#### 2.1.3. Gill Mucosal Swabs

The anterior surface of the third right hemibranch (LH3) in fish naïve to AGD was swabbed by holding open the operculum and gently rotating (×3) a disposable sterile cotton swab over the entire length of the hemibranch. The swab handle was then trimmed using sterilized dissection scissors and placed into a 1.5 mL tube containing RNAlater (1 mL) and stored at −20 °C until DNA extraction.

#### 2.1.4. Gill Histology

Gill arches were collected and processed for histological interpretation from AGD affected salmon as detailed by [33]. Briefly, gill arch tissues were fixed in seawater Davidson’s solution for 24 h before transfer to 70% ethanol prior to processing. The gill arch was dehydrated and infiltrated with wax, prior to embedding and sectioning on a microtome at 5 μm. Sections were de-waxed and stained with haematoxylin and eosin and a subset of AGD positive and AGD naïve samples (*n* = 9) were observed under a light microscope (Olympus, Hamburg, Germany) and photographed (Nikon DS-Ri2, Nikon Instruments, Tokyo, Japan).

#### 2.1.5. Water Sample Collection

A sample of the culture water was collected via filtration of pooled replicates (700 mL × 3) to make a total of ~2 L^−1^ of tank water. This was completed using a peristaltic pump (RP-100 series, Lachat Instruments, Wisconsin) that passaged water across a 0.22 µm Sterivex™ (Millipore) filter membrane to retain bacterial cells. The Sterivex™ filter chamber was then flooded with 2 mL of RNAlater solution to fix bacterial cells and then stored at −20 °C prior to DNA extraction.

#### 2.1.6. DNA Extraction and Purification

All samples underwent DNA extraction using the DNeasy Blood and Tissue Kit as per manufacturer’s protocol for animal tissue samples (Qiagen, Hilden, Germany). Samples were lysed (5 h), before washing and elution were completed. For both swab and tissue samples, total genomic DNA was assessed for yield and quality using a Nanodrop ND-1000 spectrophotometer (Life Technologies, Carlsbad, CA, USA). Samples were stored at −80 °C until sequencing.

#### 2.1.7. 16S Amplicon Sequencing

Sequencing was performed at the University of New South Wales, Ramaciotti Center for Genomics (Sydney, Australia) via an Illumina Miseq platform with 300 base pair (bp) paired end reads. The sequencing targeted the V1–V3 hypervariable region of the 16S rRNA gene in this study (“27F”AGRGTTTGATCMTGGCTCAG; and “519R”GWATTACCGCGGCKGCTG) as per the protocols in [35,36]. A total of 33 cycles were performed with normalized DNA at an average of 10 ng·µL^−1^. Samples in this study were composed of two separate sequencing runs, with both runs including a mock positive control (ZymoBIOMICS Microbial Community Standard, Zymo Research), and two negative controls (blank swab process control and blank DNA extraction laboratory control).

#### 2.1.8. Bioinformatic and Statistical Analyses

Raw Illumina amplicon sequencing data files were processed using the open-source software pipeline “Quantitative Insights into Microbial Ecology 2”QIIME2 (release 2018.8) [37]. Paired end sequences from the forward and reverse reads were merged for each sample and were denoised using the q2-dada2 plugin [38] with default parameters. Quality control including chimeric sequence removal from the dataset was completed during dada2 processing, along with subsequent removal of host DNA and exclusion of chloroplast and mitochondrial sequences. Amplicon Sequence Variants (ASV’s) were classified taxonomically using the classify-sklearn method in the QIIME2 q2-feature-classifier plugin using default parameters [39]. The SILVA 16S rRNA 99% taxonomy database release 132, [40], was used as reference sequences for taxonomic classification.

#### 2.1.9. Statistical Analysis

All statistics were performed in R project version 3.6.0, Vienna, Austria [41]. Samples were rarefied using R package QsRutils [42]. Using the Phyloseq package [43] taxonomic assignments were generated and alpha diversity indices calculated (Observed ASV’s, Shannon diversity, Faith’s phylogenetic distance). The alpha diversity metrics were analyzed via non-parametric means (Kruskal-Wallis test) and further pairwise comparisons using a Wilcoxon Test (Rank Sum Test). Beta-diversity comparisons were made via NMDS using Bray Curtis pairwise distances. Differences between groups was analyzed using the Vegan package ANOSIM [44]. Differential abundance testing was completed using the DeSeq2 package found in [45]. All figures were produced using the R package ggplot2 [46].

#### 2.1.10. Quantitative PCR

PCR assays were used to quantify the abundance of *T. dicentrarchi* and *N. perurans* in the 10 LB and 10 NLB samples from AGD affected fish. A TaqMan quantitative PCR was designed to amplify a 153 bp region of the *T. dicentrarchi* 16S rRNA gene. Specific primers and probe combinations were designed based on multiple *Tenacibaculum* species sequence alignments of 16S, obtained from the NCBI nucleotide database (Figure S1) and ASV representative sequences from the 16S amplicon sequence data. Sequence alignment from *T. dicentrarchi* type strain 35/09T (Accession number FN545354) showed 100% homology along the 153 bp fragment to ASV ID_748706 from this study, and was used to construct primer and probe design (Table 1). Primer and probe concentrations were then optimized across a dilution series from 50 to 900 nM, and the optimal primer/probe combination selected based on lowest Ct mean and variation between triplicates (Tables S1 and S2). PCR was performed in a single-plex 25 µL reaction containing 2× buffer, 50 mM MgCl, 10 mM dNTP, 10 µM forward and reverse primer, 10 µM of probe and 0.4 units of DNA Taq polymerase (Sensifast, Bioline). Each reaction contained 2 µL of normalized template DNA (30 ng·µL^−1^). PCR reactions were subjected to the following thermal cycling: 95 °C for 10 min, then 95 °C for 15 s and 60 °C for 1 min for 40 cycles, and a hold of 4 °C. A quantitative PCR assay of *N. perurans* was also performed using methods previously described by [47]. Quantitative PCR data was analyzed as a relative standard curve (Figure S2), which was produced using a cloned plasmid for both *T. dicentrarchi* and *N. perurans* amplicons. Plasmid DNA was cloned using the pGEM-T easy vector system (Promega, Madison, WI., USA), and quantified using a nanodrop spectrophotometer. To calculate 16S rRNA copies number corresponding to the cycle threshold (Ct) values, the basepair length of the amplicon fragment and vector, along with DNA concentration were submitted into a DNA-copy number calculator [48].For each target a plasmid standard curve ranging between 4.4 × 10^9^ and 0.44 copies.µL^−1^ was run on each plate and used to extrapolate the copy number within each sample as described previously [49].

## 3. Results

### 3.1. AGD Pathology

**Figure 2:**
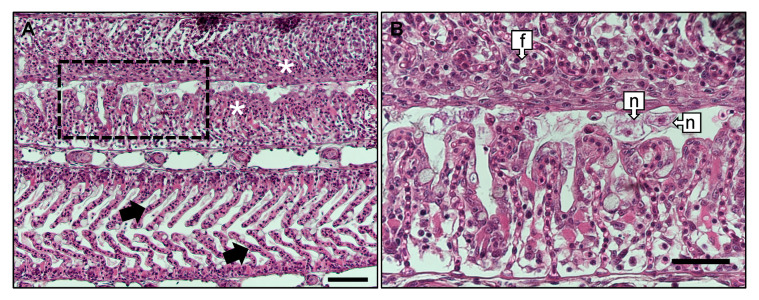
(**A**)—Gill filaments from AGD affected fish, showing anatomically normal secondary lamellae in lower filaments (black arrows), adjacent to a hyperplasic lesion induced by *N. perurans* (white asterisks), Bar = 100 µm. Inset border (dashed line box) corresponds to (**B**), at 40× magnification. (**B**)—frame subset of (**A**) showing fusion of secondary lamellae (f), and *N. perurans* trophozoites (n) with nucleus and endosymbiont present (Bar = 100 µm).

Gross clinical signs of AGD including raised multifocal lesions on the gill surface were observed in AGD affected fish (Figure 1). Macroscopic gill lesions were clearly distinguishable and were successfully biopsied from AGD affected fish. Collectively these fish had an average gross gill index of 3.30. Gill lesions indicative of AGD were not observed in salmon unexposed to *N. perurans.* Histologically, AGD affected fish displayed multifocal lamellar hyperplasia, lamellae fusion, interlamellar vesical formation and oedema (Figure 2) in close association with trophozoites of *N. perurans.*

### 3.2. 16S Amplicon Sequencing

The V1–3 region of the 16S rRNA gene was successfully amplified from all DNA samples, and sequenced as 300 bp paired end read. The number of raw and quality filtered sequence reads is provided in Supplementary Table S1. For subsequent microbiome analysis the read depth was subsetted to 4100 reads to provide even sampling effort. All sequence reads were deposited into the SRA under BioProject PRJNA649054.

### 3.3. Alpha and Beta Diversity

**Figure 3:**
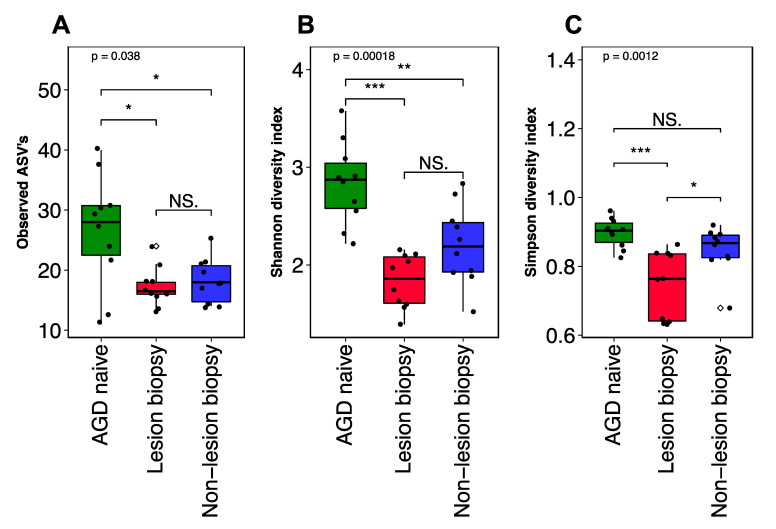
Measures of bacterial alpha diversity in naïve and AGD affected salmon gill. Mean species richness (**A**) was higher in naïve smolt than both AGD groups, whilst diversity (**B**,**C**) were highest in Naïve fish, and significantly different between Naive and LB AGD groups. Black dots represent each individual sample point, unfilled diamond shape indicates outliers. P = global significance (Kruskall Wallis), pairwise significance determined by a Wilcoxon test with *p* < 0.05, *p* < 0.01 and *p* < 0.001 represented by *, **, and ***.

Assessment of bacterial taxa richness and diversity was used to compare community structure of AGD unaffected (naïve), Lesion biopsy (LB) and Non-lesion Biopsy (NLB) smolt groups. There was a significant difference in observed ASV’s, between sample groups (KW test, *X*^2^ = 6.56, *p* < 0.05; Figure 3A), with naïve fish having higher species richness than both AGD sample groups (Wilcox; *p* < 0.05). For population diversity metrics, the Shannon species evenness (KW test, *X*^2^ = 17.21, *p* < 0.001; Figure 3B) was significantly different between two groups, specifically the Naïve group showed higher species evenness than both LB (Wilcox; *p* < 0.001) and NLB samples (Wilcox; *p* < 0.01), and the naïve vs both AGD sample groups (Wilcox; *p* < 0.001). Simpson diversity (KW test, *X*^2^ = 13.44, *p* < 0.01; Figure 3C) was also highest in the naïve group, which was not different to NLB, but significantly higher than LB (Wilcox; *p* < 0.001). The NLB group also proved to be significantly different to the LB group (Wilcox; *p* < 0.05). Overall these results point to a more abundant and diverse bacterial community present within the naïve samples and a decreasing bacterial diversity within the AGD lesion site.

**Figure 4:**
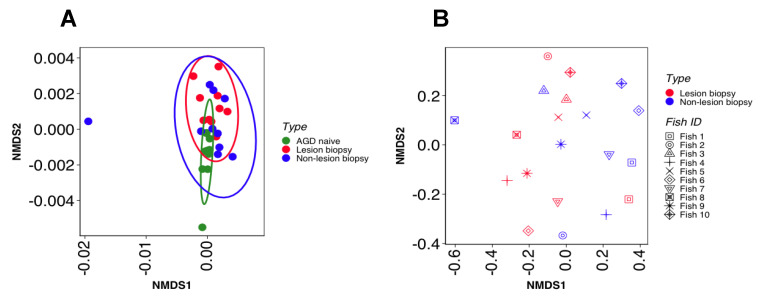
Beta diversity analysis using non-metric multidimensional scaling of Gower distance dissimilarity of (**A**), all sample types (stress = 0.17) and Bray Curtis ranked distance (**B**), LB and NLB samples denoting individual fish by shape (stress = 0.21). These data indicate that groupings between sample type were distinct, and that fish-to-fish variation of the LB and NLB groups was high.

Comparison of between-sample variance of bacterial communities by group was investigated by non-metric multidimensional scaling plots using ranked distance metrics (Figure 4A). Statistical analysis of similarity (ANOSIM) between lesion biopsy groups, demonstrated a significant difference between bacterial community composition of naïve, LB and NLB groups (ANOSIM, *p* < 0.05). Between AGD sample comparison (Figure 4B) indicates some overlap and variation between the LB and NLB groups, where there was appreciable interspecific (fish-to-fish) variation. (Figure 4B).

### 3.4. Taxonomic Assignment and Composition

**Figure 5:**
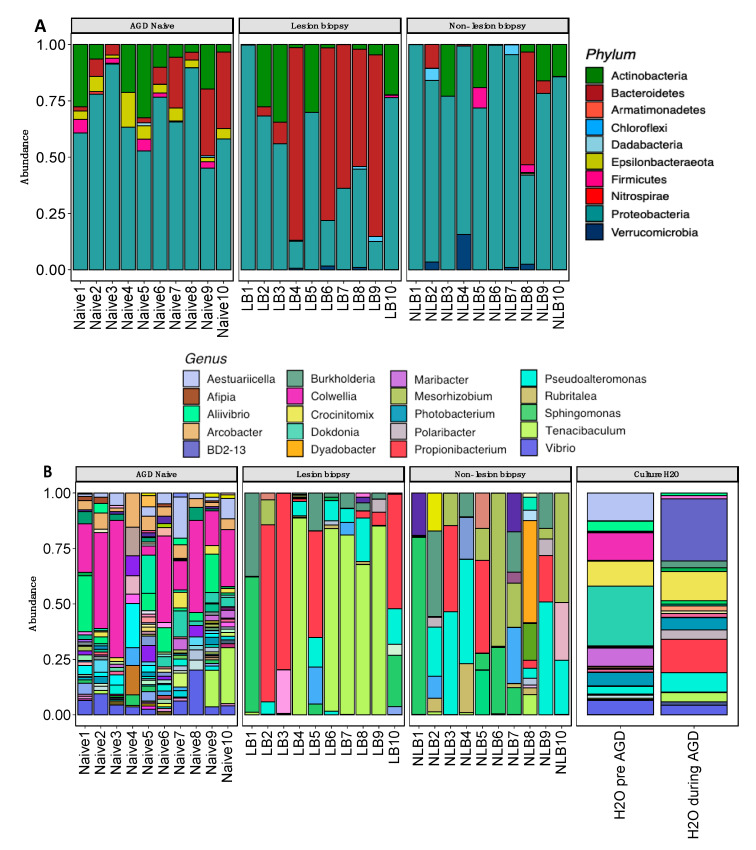
Microbial abundance of samples in this study. Plots show relative abundance (%) for Naïve, LB and NLB and fish at the phylum level (**A**) and Sterivex water samples at the genus level (**B**) level. Overall diversity of bacterial communities in the naive fish were higher, where AGD affected fish identified lower numbers of taxa with higher abundances.

A total of 4856 ASVs were identified across our samples and were assigned to 38 phyla, 202 order, 299 classes and 471 genera. Differences in taxa abundance was observed between the US, LB and NLB samples. At the phylum level, there was a shift in dominant phyla with an increase in *Bacteriodetes* abundance (37.2%), which was increased from Naïve (11.3%) and NLB (6.3%) sample groups (Figure 5A). The predominant phylum for Naïve and NLB remained *Proteobacteria* (68.1% & 81.6%), which was reduced in LB samples (49.6%) (Figure 5A). *Actinobacteria* was consistent across all three groups, but highest in LB samples (12.2%). Several Phylum were present at <1% abundance throughout. At the genus level, considerable diversity was observed across the naïve samples, with a higher number of low abundance assignments, the profile of which aligned closely to the source water sample at that timepoint (Figure 5B). The NLB sample group had a lower number of genus taxa than the AGD naïve group, but was more diverse than the LB samples. In the LB samples we observed a high mean abundance (70.7%) of a single ASV classified to the genus *Tenacibaculum* in five of the ten samples, and lower abundance in all other LB samples (Figure 5B). In contrast this ASV was significantly less abundant or absent in the naïve and NLB samples, specifically being absent in four of the ten NLB samples, with a mean abundance of 0.83% in remaining samples and only one sample > 1%. Closer inspection of the taxonomic classification of this ASV using a global alignment tool, Genbank (NCBI), demonstrates a 100% sequence identity to *T. dicentrarchi*. Source water collecting during the AGD episode also shared some key taxa with the biopsy samples, notably *Pseudoaltermonas*, *Propionibacterium* and *Tenacibaculum*.

**Figure 6:**
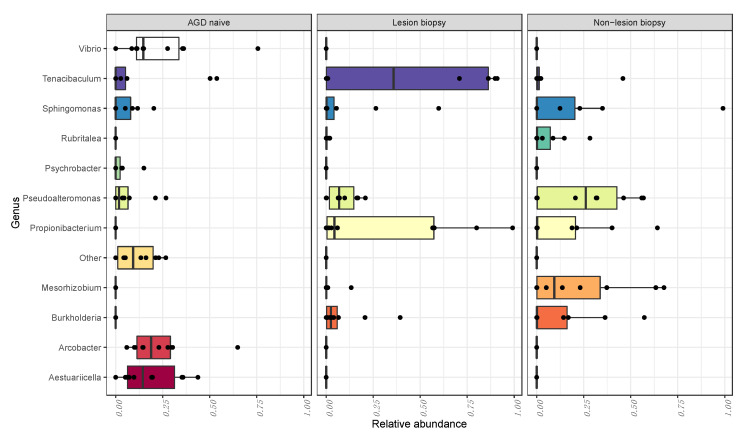
Top 11 genus differentially expressed between naïve, LB and NLB groups (DESeq2). The differential analysis was compared between all groups, with an adjusted *p* value of (*p* < 0.001). Data shows that the taxa *Propionibacterium* and *Tenacibaculum* have a significantly stronger association to AGD lesions than in other groups.

The abundance of ASVs were agglomerated to the genus level and compared between groups using pairwise comparisons within a negative binomial general linear model. A total of 11 ASVs were deemed significantly differentially expressed across our dataset. A significantly higher abundance of ASV ID_748706, which was classified as *T. dicentrarchi*, was observed in the LB compared to the NLB and naïve groups (Figure 6).

From the taxonomic assignments to the genus level, we compared taxa which were differentially abundant between sample groups. Based on Figure 6, the analysis identified 11 assigned taxa (in addition to ‘Other’ grouped as the < 1% abundant taxa) which were positively or negatively expressed between groups. *Tenacibaculum* and *Propionibacterium* were the dominant genus with the AGD affected gill tissue (LB group). Several taxa including *Arcobacter*, *Vibrio* and *Aestuariicella* were identified as positively expressed in the naïve group, but were in negligible numbers in both AGD positive sample groups.

### 3.5. Quantitative PCR

**Figure 7:**
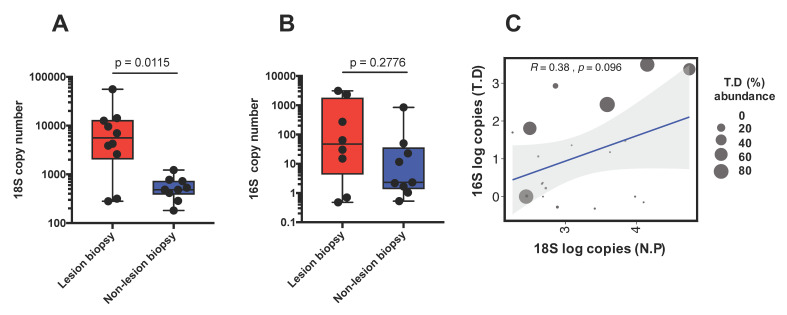
(**A**)—18S gene copy number, and (**B**)—16S gene copy number (**B**) for both *N. perurans* and *T. dicentrarchi* qPCR assays respectively, comparing LB and NLB sample groups (*p* value = Mann-Whitney non parametric test). (**C**)—Pearson correlation analysis of log transformed copy numbers of LB samples (overlaid with *Tenacibaculum* % abundance), suggesting a moderate positive relationship between *N. Perurans* and *T. dicentrarchi* loads within AGD lesion sites.

A quantitative TaqMan PCR was developed and optimized for *T. dicentrarchi.* The optimized primer and probe concentrations were 100 nM (forward and reverse) and 50 nM respectively. The limit of detection (LOD) of assay was determined to be greater than 0.11 plasmid copies (Table S3), with an amplification efficiency of 2.1. The abundance of *T. dicentrarchi* and *N. perurans* was then examined in the lesion and non-lesion biopsies using these quantitative PCR assays. A relative standard curve analysis was performed and abundance presented as 18S or 16S copies for *N. perurans* and *T. dicentrarchi*, respectively. Lesion biopsies had a significantly (*p* = 0.0115) higher abundance of *N. perurans* 18S copies compared to non-lesion biopsies (Figure 7A). In agreement with the microbiome analysis we also observed an increased abundance of *T. dicentrarchi* 16S copies in the lesion biopsies (Figure 7B).However due to the considerable fish-to-fish variation, the difference in *T. dicentrarchi* 16S copies between lesion and non-lesion biopsies was not statistically significant. A positive, but non-statistically significant correlation between *N. perurans* 18S and *T. dicentrarchi* 16S copies was observed (Figure 7C; Pearson; *R* = 0.38, *p* = 0.096). 

## 4. Discussion

AGD is a costly and detrimental ectoparasitic infection of salmonid species. Whilst the causative agent of the condition has been explicitly confirmed, only a few studies have examined the bacterial community in fish affected with AGD. The potential for host microbial dysbiosis that could influence AGD progression or increase disease vulnerability is to date largely unexplored. Understanding the bacterial community composition before and during an AGD episode as well as discrete recruitment to AGD affected gill microhabitats may facilitate further understanding *of N. perurans* pathogenesis.

Results from this study demonstrate that the bacterial community of the gill can be significantly altered following challenge with AGD. The observed bacterial taxa richness and diversity of AGD challenged fish was lower in biopsy samples containing branchial lesions. In contrast, salmon unexposed to *N. perurans* showed significantly higher bacterial richness and a more diverse and even community composition. Dysbiosis of teleost fish mucosal surfaces have been previously identified for a range of scenarios, whereby the commensal microbiome has been perturbed or compromised during infection. An in vivo challenge trial [7], demonstrated that the skin microbiota of Atlantic salmon infested with the sea louse (*Lepeophtheirus salmonis*) was susceptible to colonization from known pathogenic genera, including *Vibrio*, *Flavobacterium*, *Tenacibaculum* and *Pseudomonas*. It was also observed that over time, bacterial species’ richness declined in swab samples from the whole skin surface. Similarly, dysbiosis of the microbial community was also evident in Atlantic salmon skin following salmon alphavirus (SAV) infection [50] where bacterial community derived from a discrete skin sample indicated a global decrease in bacterial richness and diversity from low and high dose SAV groups at 14 dpi. Changes in the bacterial profiles were characterized by a decreasing abundance of *Proteobacteria*, and increasing colonization of opportunist pathogens including *Flavobacteriaceae, Streptococcaceae* and *Tenacibaculum*. While it appears clear that both parasitic and viral infections can significantly reduce the microbial diversity in affected tissues, the consequence of dysbiosis upon disease pathology and the host response remains unclear.

Microbial imbalance is commonly associated with pathogen insult, but disease treatment options can also significantly alter the microbial community, and potentially contribute to disease development and severity. Channel catfish (*Ictalurus puntactus*) treated with potassium permanganate led to a bacterial dysbioses of the skin and gills in vivo [51]. Subsequent bacterial challenge of *Flavobacterium columnare* then resulted in significantly lower community diversity, and increased mortality for treated/challenged fish in comparison to non-treated/challenged counterparts. Other examples of gill co-infections and subsequent increased disease susceptibility are seen in complex gill disease (CGD). This is a gill condition associated with a number of different known agents, including *N. perurans*, Candidatus *Piscichlamydia salmonis, Desmozoon lepeophtherii*, salmon gill poxvirus and Candidatus *Branchiomonas cysticola* [52]. It has been suggested that the primary pathogen/s compromise host immunity that leaves the gills vulnerable to colonization by opportunistic pathogens [53,54]. In the case of AGD, downregulation of critical immune pathways, including the major histocompatibility complex (MHC), has been observed within AGD branchial lesions [55]. This research suggests that immune function within the AGD affected tissue may be compromise which may ultimately contribute to the successful colonization of pathogen bacterial taxa. Overall, data from our study corroborates with other published works demonstrating that the profiles of bacterial communities in gills are susceptible to alteration following pathological challenge.

Differential abundance testing showed significantly different genera between sampling groups. NLB samples contained a significantly higher abundance of *Pseudoalteromonas* and *Mesorhizobium*, whilst interestingly, branchial lesion biopsies had a much higher proportion of *Tenacibaculum* and to a lesser extent *Propionibacterium*. It has been shown that Atlantic salmon affected by AGD (with branchial lesions) are functionally compromised, with significantly lower aerobic scope, as well as decreased capacity to regulate homeostasis [56]. These impacts, amongst others have downstream effects on appetite and immune vitality [57], and therefore waste excretion. Taxa which perform specialist biological functions readily colonize the healthy gill surface [9,58]. For example, nitrogen fixing bacteria were linked to the expression of nitrogenous wastes at the gill surface in common carp (*Cyprinus carpio*) and zebrafish (*Danio rerio*) [59]. In the instance of AGD hyperplasia where cellular tissue has lost excretory function, it is possible that this would ultimately impact the bacterial taxa from this functional group of taxa colonizing such areas, affecting overall diversity. In the current study, NLB samples contained significant proportions of known nitrifying genera (*Mesorhizobium* and *Burkholderia*), which were not as abundant in LB samples specifically. In turn, *Propionibacterium*, a gram positive anaerobic taxa group, is commonly associated with skin and gland habitats, where they are able to effectively metabolize carbohydrates and carbon dioxide [60,61]. The most significant change in bacterial community composition in AGD affected gills was the presence of an ASV classified to *Tenacibaculum dicentrarchi*, which was highly abundant (52.3–85.8%) in 50% of the sampled fish, and strikingly was differentially abundant between LB and NLB groups. This ASV was present at low to negligible levels in some of the naïve and NLB samples (along with *T. mesophilum* in the naïve group), though not in the high abundances seen in the LB group. Similarly, there was a negligible quantity of *T. dicentrarchi* present in the source tank water sampled both before and during the AGD challenge. Amplicon sequence data was supplemented by application of a *T. dicentrarchi* specific qPCR assay, confirming presence of the species in AGD affected fish. This assay also confirmed the presence and high quantity of 16S gene copy numbers relating to *T. dicentrarchi* biopsies containing lesions suggesting that *T. dicentrarchi* were in greater abundance with diseased gill tissues in of AGD affected salmon. Review of the full annotated genome for *T. dicentrarchi* in [62] demonstrates that 10 copies of the 16S gene are present. As the LB samples had approximately seven times the mean 16S copy number compared to the NLB group (728 to 104 copies·µL^−1^), this indicates that the approximate average of *T. dicentrarchi* bacterium per sample have been at a concentration of ~7280 bacterium per lesion biopsy sample.

*Tenacibaculum* have been identified as detrimental opportunist pathogens [63], and proliferation of these taxa may be increased in the presence of functionally impaired gill tissue. *Tenacibaculum* (primarily belonging to *T. maritimum, T. dicentrarchi* or *T. finnmarkense*) are a group of emerging pathogens in global aquaculture, with several notable disease-causing species [64,65,66,67]. A naturally occurring marine bacterium, it is usually horizontally transmitted in the water column and rapidly develops into an ulcerative disease of the skin, mouth, fins and gills, where extensive mats of gram-negative bacteria can cause significant tissue erosion and mortality [62,63,68]. Protozoan-bacterial coinfections have also been previously documented. At several Canadian farm sites, Arctic char (*Salvelinus alpinus*) and rainbow trout (*Oncorhynchus mykiss*) infected with visible bacterial gill disease (*Flavobacterium branchiophilum*) were concomitantly affected with nodular gill disease tentatively attributed to a secondary amoebic infestation [69]. In a previous laboratory challenge of Atlantic salmon with *Tenacibaculum maritimum* which were sub-clinically affected by AGD, a noticeable increase was observed in mortality of co-infected fish compared to those solely affected by AGD [70]. A longitudinal on-farm survey by Downes et al. [71] assessed several key salmonid pathogens, finding simultaneous qPCR pathogen load increases of *N. perurans* and *T. maritimum* on the gills of salmon during the grow out period. Confirmation of successful AGD induction in this study was completed using several diagnostics. Affected fish used in this study were moderately to severely affected by AGD based on individual gross gill scores [72]. Data from qPCR in this study indicates that both *N. perurans* 18S and *T. dicentrarchi* 16S copy numbers simultaneously increased at lesion sites and showed a moderate positive relationship between loads of each organism. The presence of *T. dicentrarchi* on branchial gill lesions found in this study may have arisen in a secondary manner similar to other parasitic infections. The skin microbiome of sea louse infected Atlantic salmon had higher abundances of known pathogenic taxa, including *Tenacibaculum* [7]. However, it is possible that the colonization of branchial lesions by *T. dicentrarchi* may be analogous to *T. maritimum* being conveyed in a trojan manner by an intermediate vector. This parallel has previously been drawn between *T. maritimum* and a jellyfish species *Phialella quadrata*, that caused damage to Atlantic salmon gills and subsequently followed by an outbreak of tenacibaculosis [73]. Bacterial sequence data from both jellyfish manubrium (mouth) and salmon gill samples showed a near identical resemblance of *T. maritimum,* indicating that the bacterium may be passing from the jellyfish to the gill, not as a separate opportunistic infection. Tenacibaculosis has also been characterized in Atlantic salmon net pen cleaner fish, lumpsucker (*Cyclopterus lumpus*) [74], also demonstrating a horizontal transmission source for pathogenesis. Precedence exists for amoebae to play the role of a transmission vector [75], but this has not been documented for *N. perurans.*

Focal superficial necrosis associated with attachment of *N. perurans* trophozoites has been observed upon associated gill lesions [27,28], implicating a role for extracellular products (ECPs). ECPs produced by *N. perurans* have been associated with the destruction of cell monolayers in vitro as potential virulence factors for pathogenesis of *N. perurans* and the *Tenacibaculum* genus [25,63]. Epithelial disintegration of the cellular epithelium induced by potent exotoxins produced by the bacteria is an important clinical sign of infection by *Tenacibaculum* sp. [64,76]. Given amoebic/bacterial associations and symbioses are well documented [77,78] it may be possible that *N. perurans* could utilize bacterial ECPs as a virulence factor, although this requires further investigation.

In conclusion, this study found a significant decrease in bacterial diversity in AGD affected gill tissues compared to gill mucus from fish naïve to AGD. Biopsy samples with branchial gill lesions were also lower in bacterial diversity compared to adjunct tissues without visible lesions. Additionally, this study observed a potential association of *T. dicentrarchi,* a known agent of *tenacibaculosis,* with branchial gill lesions induced by infection with *N. perurans*. *Tenacibaculum* sp. and potentially other virulent bacteria associated with *N. perurans* may play a role in the development of amoebic gill branchialitis.

## Figures and Tables

**Table 1:** Nucleotide sequences of primers and probes both designed and used in this study, for the real time PCR detection of *T. dicentrarchi* and *N. perurans* DNA fragments.

Assay (Gene)	Primer	Sequence (5′-3′)	Length	Ref
*T. dicentrarchi* *(16S rRNA)*	FWD	TAACATTATGCTTGCATAGATGACGA	26 bp	*Current study*
	REV	AGCCTTGTGATAATTTGTAAATACCCATG	29 bp	
	Probe	FAM-CCTTTAGAAATGAAGATTAATACTCCATAATGTAGTGATTCGG-MGB	43 bp	
*N. perurans* *(18S rRNA)*	FWD	AAAAGACCATGCGATTCGTAAAGT	24 bp	Downes et al. [47]
	REV	CATTCTTTTCGGAGAGTGGAAATT	24 bp	
	Probe	FAM-ATCATGATTCACCATATGTT-MGB	20 bp	

## Supplementary Materials

The following are available online at https://www.mdpi.com/2076-2607/8/8/1189/s1, Figure S1. Sequence alignment of partial 16S rRNA genes from nine notable, Figure S2. Standard curve obtained from 10-fold dilutions of T. dicentrarchi plasmid, Table S1. Tenacibaculum dicentrarchi quantitative PCR validation primer concentration, Table S2. Validation of probe concentration in Tenacibaculum dicentrarchi quantitative PCR assay, Table S3. Limit of detection (LOD) for T. dicentrarchi qPCR assay.
