# Supplementary material for: Bacteriomic Profiling of Branchial Lesions Induced by Neoparamoeba perurans Challenge Reveals Commensal Dysbiosis and an Association with Tenacibaculum dicentrarchi in AGD-Affected Atlantic Salmon (Salmo salar L.)
Source: Microorganisms. 2020 Aug 5;8(8):1189. doi: 10.3390/microorganisms8081189 (PMC7464746; doi:10.3390/microorganisms8081189)
Supplement: Supplementary file 1 [file microorganisms-08-01189-s001.pdf]

Supplementary:

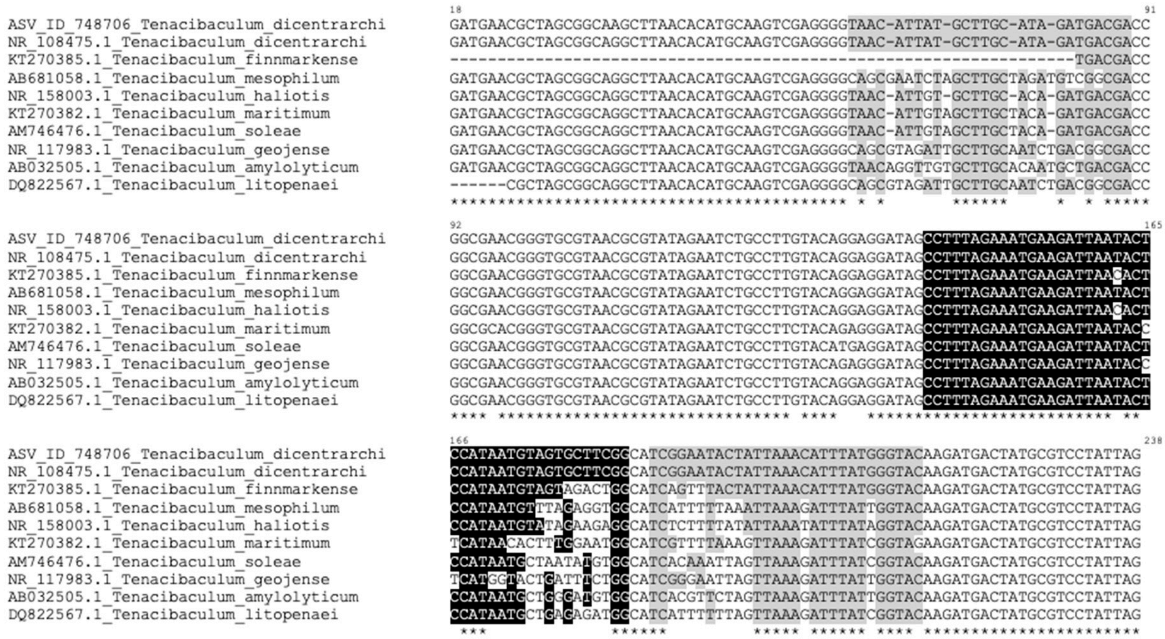

Figure S1. Sequence alignment of partial 16S rRNA genes from nine notable *Tenacibaculum* species (including accession number), and ASV sequence from the current study. The primer (grey shaded) and probe (black shaded) selections were based on unique conserved areas between the species sequences. Asterisk indicates no difference in nucleotide between species at each base position.

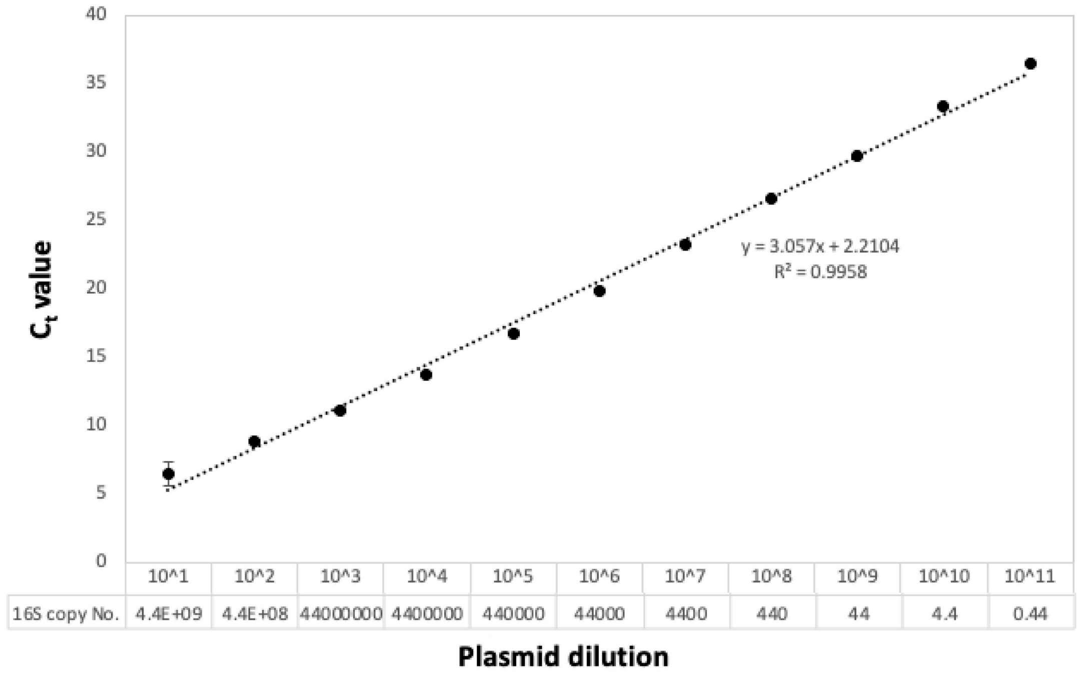

Figure S2. Standard curve obtained from 10-fold dilutions of *T. dicentrarchi* plasmid. At each dilution point the Ct value was plotted against dilution in triplicate.

Table S1. *Tenacibaculum dicentrarchi* quantitative PCR validation primer concentration. Combinations of primer concentrations were assessed using a fixed probe concentration of 100 nM.

	Forward and Reverse Primer Combinations (nM)								
	100F/100R	*50F/300R*	*50F/900R*	*300F/50R*	*300F/300R*	*400F/400R*	*300F/900R*	*900F/50R*	*900F/300R*
**Mean Ct**	**20.18**	20.08	20.00	20.58	20.11	20.24	20.11	20.65	20.92
**SD**	**0.03**	0.13	0.05	0.08	0.08	0.09	0.12	0.02	0.14

Table S2. Validation of probe concentration in *Tenacibaculum dicentrarchi* quantitative PCR assay. Various probe concentrations were assessed using a selected primer concentration of 300 nM (Forward) and 300 nM (Reverse).

	25nM	***50nM***	*75nM*	*100nM*	*125nM*	*150nM*
**Mean Ct**	20.04	**19.71**	19.76	20.20	19.95	19.62
**SD**	0.07	**0.08**	0.50	0.13	0.12	0.09
	175nM	*200nM*	*225nM*	*250nM*	*275nM*	*300nM*
**Mean Ct**	19.29	19.60	19.47	19.86	19.50	19.30
**SD**	0.28	0.14	0.43	0.23	0.33	0.14

Table S3. Limit of detection (LOD) for *T. dicentrarchi* qPCR assay.

T. Dicentrarchi LOD					
16S Copy No.	*Ct 1*	*Ct 2*	*Ct 3*	*Mean*	*SD*
**0.22**	37.34	37.64	37.34	37.44	0.17
**0.11**	38.05	38.10	37.54	**37.90**	0.30
**0.05**	Und.	38.23	38.44	38.34	0.14
**0.02**	38.34	Und.	Und.	38.34	n/a
